# Household survival and resilience to food insecurity through the drip irrigation scheme in dry rural areas

**DOI:** 10.4102/jamba.v13i1.985

**Published:** 2021-03-25

**Authors:** Faith R. Chidavaenzi, Adrino Mazenda, Ntobeko Ndlovu

**Affiliations:** 1 Institute of Development Studies, Faculty of Commerce, National University of Science and Technology, Bulawayo, Zimbabwe; 2 School of Public Management and Administration, Faculty of Economic and Management Sciences, University of Pretoria, Pretoria, South Africa

**Keywords:** drip irrigation, food security, household resilience, Chidzadza irrigation scheme, Zimbabwe

## Abstract

Increasing food production by developing small-scale irrigation schemes is a requirement for tackling household food insecurity. Strategies, such as the World Vision, Enhancing Nutrition, Stepping Up Resilience and Enterprise, have been established to enhance food availability in the drought-prone Burirano Ward 4, Chipinge, Zimbabwe, through the drip irrigation intervention. This study analysed the extent to which the drip intervention has increased food production, abilities, income and nutrition of households. Consequently, the key factors impacting the performance of the drip irrigation scheme were assessed. The study utilised a mixed-method convergent parallel design, drawing from semi-structured questionnaires administered on a census of 40 household beneficiaries as well as a focus group discussion of five key informants directly linked to the Chidzadza irrigation scheme, Burirano Ward 4, Chipinge, Zimbabwe. The findings show that the drip irrigation scheme significantly increased households’ food production abilities, nutrition and income. The main factors responsible for the success of the drip irrigation scheme are cheap labour from household members and agriculture extension support. Issues that prevent the success of the scheme include erratic rain supplies and damaged water pipes. Strategies to increase household food production through the drip irrigation scheme include maintenance of water pipes, an increase in water catchment areas and water availability through solar-powered borehole systems.

## Introduction

About 842 million people in the world do not have enough to eat, which is attributable to acute poverty, whilst up to 2 billion people are food insecure (reliefweb 2018). Zimbabwe experiences irregular rainfall patterns, ranging from the El Niño-induced consecutive severe droughts in 2014/2015 and 2015/2016 to the La Nina-induced above-normal rains and flooding in 2016/2017. This irregular rainfall renders it impossible for societies to adjust crop patterns in reaction to climate change, adversely impacting agricultural production and submerging communities into food insecurity and absolute poverty.

Developing irrigation schemes is a requirement for tackling household food insecurity in dry areas. Irrigation increases the capacity to produce food in severe drought conditions and expands possibilities for agriculture production and crop diversification (Narayanamoorthy & Devika 2018; ZimVAC 2018). Small-scale irrigation consisting of sprinkler/overhead irrigation, flood irrigation, furrow irrigation and drip irrigation systems has been identified as ideal for supplementing rain-fed agriculture and ensuring food security, especially in low-rainfall areas (Mango et al. 2018; Riesgo et al. 2016). Irrigation systems alleviate poverty by increasing production, income and nutrition. This is because of surplus sales and crop diversification (Bjornlund, Van Rooyen & Stirzaker 2017; Passarelli et al. 2018; Tefera & Cho 2017).

The drip irrigation system is favoured in dry regions because of its effective water use capability as well as lower maintenance requirements and the potential to function on any gradient (Adamala 2016; FAO 2016; Lozano et al. 2020). However, the device has large maintenance costs and can operate poorly if users are inexperienced or unaware of simple maintenance procedures, such as the cleaning of filters (Adamala 2016; Ruban et al. 2020).

World Vision, through the Enhancing Nutrition, Stepping Up Resilience and Enterprise (ENSURE)^1^ initiative, sponsors the Chidzadza Drip Irrigation Scheme to increase agricultural production in the Burirano Ward 4, Chipinge District, Zimbabwe. By assessing this scheme, this study addresses the following research questions:

- To what extent has the drip irrigation scheme increased food production abilities, nutrition and income of households?
- What are the key factors impacting the performance of the drip irrigation system?
- What are the possible strategies that can be adopted to increase food production, through the drip irrigation system?

The study expands on previous studies (Mango et al. 2018; Mhembwe, Chiunya & Dube 2019; Mtonga 2014; Peter 2011) which argue that plenty of financial resources and support were invested in Zimbabwean rural irrigation schemes to increase food production and consequently promote sustainable food security. Despite these efforts, household food insecurity still prevails, most prevalently from the production (availability) point of view.

This study is structured as follows: after the introduction, food security and irrigation are conceptualised in the Conceptual Framework section, which is followed by Materials and Methods, Results and Analyses, and Conclusion sections.

## Conceptual framework

The capacity for irrigation to increase food production depends on a variety of factors such as the water reservoir (i.e. aquifers, underground water and safe water), the specific water availability (i.e. single season, intermittent or full season), the type of irrigation system (i.e. drip, sprinkler or furrow), size of the system (large scale and small scale) access to farm inputs (i.e. land, credit, seeds, fertiliser and labour), the socioeconomic characteristics of the household and the administrative laws regulating access to and management of the irrigation systems (Domènech 2015; Oluwasegun et al. 2020).

Numerous studies have shown that the implementation of irrigation technology will play an important role in poverty reduction, food and nutrition safety, and household income (Bedru et al. 2020; Burney & Naylor 2012; Rosegrant, Ringler & Zhu 2009). Irrigation will allow subsistence farmers to engage in year-round production, increase yields and boost food security and livelihoods (Smith, Alderman & Dede 2006; Ye, Han & Liu 2019). In dry tropical regions, households experience chronic shortages of vegetables and fruit. This has a direct impact on household nutritional safety. Access to irrigation will enable smallholder farmers to grow varying crop varieties and sell the surplus to the local market (Bedru et al. 2020). The use of small-scale irrigation technologies encourages crop diversification and greatly improves land returns (Burney & Naylor 2012; Montazar, Cahn & Putman 2020). More recent literature describes unique mechanisms (impact pathways) connecting irrigation to beneficial food and nutritional outcomes (Passarelli et al. 2018).

**FIGURE 1:**
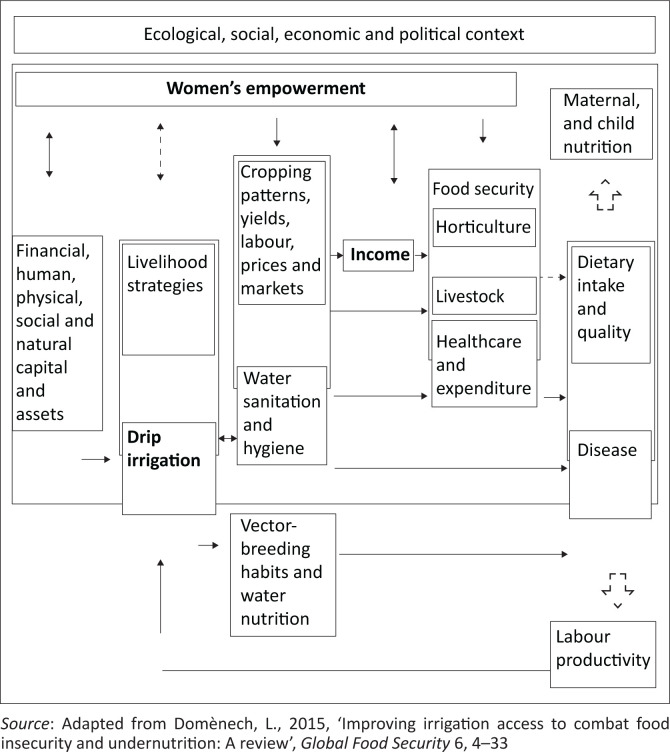
Conceptual framework.

The drip irrigation, which is utilised in the Chidzadza irrigation scheme, is the most effective irrigation system for small-scale farming in semi-arid areas (FAO 2016; Montazar et al. 2020; Postel et al. 2001). Despite high maintenance and start-up costs, the system allows the optimal use of water resources to achieve maximum crop yields. The conceptual framework for drip irrigation, food production and nutritional outcomes is shown in Figure 1.

Figure 1 shows the five main impact pathways linking the drip irrigation to food production, nutrition and health outcomes. The pathways are as follows:

- Irrigation as a source of increasingly diverse food (through improved farm production and crop diversification)
- Irrigation as a source of income (from market sales and job creation)
- Irrigation as a water source delivering multi-purpose water services [water sanitation and hygiene (WASH), animal husbandry and aquaculture]
- Irrigation as a vector-breeding ecosystem and a source of water contamination (from fertilisers, herbicides and pesticides)
- Irrigation as an access point for women’s empowerment (through improved asset ownership and resource control) (Domènech 2015; Ruban et al. 2020).

## Materials and methods

### Study area

Zimbabwe is divided into five regions, with regions four (IV) and five (V) often experiencing dry weather conditions. Regions IV and V experience low-rainfall patterns ranging from 450 mm – 800 mm to < 450 mm. Thus, the production of food solely through rain-fed development in rural communities in these regions is ineffective (Mhembwe, Chiunya & Dube 2019; Nhundu & Mushunje 2012). Chipinge, on which the Chidzadza irrigation scheme is based, is in region IV and receives 450 mm – 650 mm of annual rainfall. The region has been considered semi-extensive and suitable for farm systems based on livestock, resistant fodder crops, forestry and wildlife (FAO 2019). Moreover, the area has a 40% – 65% chance of rainfall which occurs between October and April. It takes between 100 and 135 days to grow crops, with yields often not sustainable because of the greyish-brown sands and sandy loams derived from granite rocks. These soils have low water-holding capacity and often cannot sustain irrigation systems such as the furrow and the overhead irrigation types (FAO 2019). Chidzadza irrigation scheme was developed through the World Vision ENSURE food resilience programme, with the goal of increasing food production in areas vulnerable to drought. The scheme lies in Ward 4, Burirano, District of Chipinge, Manicaland Province, Zimbabwe.

### Methods

The study employed a mixed-method approach drawing on the convergent parallel design. A mixed-method approach is used in social enquiry to explore a phenomenon, using two or more methods, processes and philosophies. The two overarching reasons for employing this approach in research relate to the ability of the methods to provide accurate answers to all research questions in all situations and that more than one method in most situations is likely to produce a better and more complete picture about the problem than a single method alone (Kumar 2014:20, 25). Convergent parallel design equally prioritises both quantitative and qualitative research methods. The design entails collecting numeric and text data concurrently during the same phase of the research process, independently analysing databases and only merging the two sets of results for the overall interpretation and drawing the conclusion.

The quantitative approach draws on a semi-structured questionnaire administered on a census of 40 household beneficiaries to the drip irrigation scheme. The beneficiaries were selected based on the World Vision ENSURE criteria of previous farming experience and being economically active. A questionnaire allows the respondent to focus on significant items, in a written form and with standardised instructions to enhance uniformity in the recording of responses necessary for quantitative analysis (Kumar 2014:181). Structured data from the semi-structured questionnaires were used in the quantitative analysis. Manipulation of data was performed using the Statistical Package of Social Sciences (SPSS). Presented in the analysis are the descriptive statistics on data demographics and impact assessment utilising the Mann–Whitney *U* test and Kruskal–Wallis *H* non-parametric tests. The Mann–Whitney *U* test compares the differences between two independent groups when the dependent variable is continuous or ordinal and non-normally distributed. The Kruskal–Wallis *H* test compares the differences between two independent samples of equal or different sizes (Siegel & Castellan 1988).

The qualitative approach draws on a triangulation of open-ended responses from the structured questionnaire discussed in the quantitative phase and the focus group discussion (FGD) on purposively selected experts and external stakeholders to the drip irrigation scheme. The FGD constituted of five key informants, experts from agriculture rural extension (AREX), World Vision ENSURE, village head, a ward councillor and the district administrator. The use of the FGD allowed for the encouragement of an intimate group in an accepting environment that enables respondents to share their thoughts, insights and opinions without the fear of judgment. The FGD gives room to discover the perceptions and experiences of different individuals in a particular study. Moreover, it provides the opportunity for the researcher to receive multiple opinions and a group consensus on key issues in a shorter time frame (Satterfield 2000). Thematic analysis is carried out on the semi-structured questionnaire’s open-ended responses and FGD to draw emerging themes on the challenges facing the implementation as well as operations of the drip irrigation scheme. Moreover, possible solutions are concluded on how the scheme could be utilised effectively in increasing food production.

### Ethical considerations

Ethical approval to conduct the study was obtained from the Department of Development Studies, National University of Science and Technology, Zimbabwe (reference no. NO1518602F – 1518602F).

## Results and analyses

This section presents the study findings drawn from a census of 40 semi-structured questionnaires administered to the irrigation scheme beneficiaries (quantitative analysis). Consequently, an FGD of five key informants directly involved in the irrigation scheme as well as open-ended questions from the semi-structured questionnaire (qualitative analysis) will be discussed.

### Quantitative analysis

This section provides the quantitative analysis of the responses from the semi-structured questionnaire. The first section provides descriptive statistics on the demographic characteristics of the study participants. The second provides quantitative analysis on the impact of drip irrigation on food production, using the Mann–Whitney *U* and the Kruskal–Wallis *H* tests.

#### Demographic characteristics

This section presents the demographic characteristics of the 40 household heads obtained from the semi-structured questionnaires. The first part presents the gender composition of the household heads, followed by age and education status as the key determinants in the functionality of the irrigation scheme.

**Gender of the household head:** In meeting the World Vision ENSURE, gender issues have to be considered in redressing the inequalities in society. Table 1 presents the gender composition of household participants in the irrigation scheme.

**TABLE 1:** Gender of the household head.

Gender	Frequency	%	Valid %	Cumulative %
Male	18	45	45	45
Female	22	55	55	100

**Total**	**40**	**100**	**100**	**-**

As shown in Table 1, a total of 22 (55%) respondents were female and 18 (45%) were male. This shows a demographic structure where the gender issues are considered in the selection of the study participants.

**Age of household head:** Utilising an economically active population in pilot projects is important in correctly measuring the project outcomes. Table 2 shows the age distribution of the household heads to determine their position on the 15–64 economically active population categories of the International Labour Organization (ILO 2002).

**TABLE 2:** Age of the household head.

Age group	Frequency	%	Valid %	Cumulative %
18–25	3	7.5	7.5	7.5
26–35	9	22.5	22.5	30
36–45	13	32.5	32.5	62.5
46–55	10	25	25	87.5
56+	5	12.5	12.5	100

**Total**	**40**	**100**	**100**	**-**

Table 2 shows that all households involved in crop production on the irrigation scheme are economically active; hence, they are capable of utilising the proceeds from the irrigation scheme fully.

**Education:** The level of education determines the level of assimilation of technical skills required to maintain the system. Moreover, these levels can also determine the ability to practice productive farming as taught by AREX. Table 3 shows the literacy level of the household heads.

**TABLE 3:** Level of education.

Education level	Frequency	%	Valid %	Cumulative %
Primary education	7	17.5	17.5	17.5
Secondary education	33	82.5	82.5	100

**Total**	**40**	**100**	**100**	**-**

Table 3 shows that the majority of the household heads/farmers have completed the ordinary-level certificate of their education qualification. Completion of the certificate is the evidence that the farmers can learn and apply the technicalities involved in the irrigation programme.

#### Ability to meet household food requirements

Prior to the introduction of the drip irrigation scheme, household farming was based on seasonal rain, which was unsustainable in meeting their household food requirements. Table 4 shows the crop yields prior to and after the drip interventions.

**TABLE 4:** Crop yield before and after drip intervention.

Crop yield	Crop type	*N*	Minimum	Maximum	Mean	Standard deviation
Before drip	Maize	40	30	765	310	149
	Beans	40	0	165	65	56
	Covo	40	10	45	22	7
	Tomatoes	40	0	175	62	85
With drip	Maize	40	100	1300	607	253
	Beans	40	50	380	182	75
	Covo	40	20	65	35	9
	Tomatoes	40	22	350	206	83

Note: All yields are converted to kilograms (kg) (1 bundle of covo = 1 kg).

It can be observed from Table 4 that production increased after the administration of the drip intervention strategy. The greatest yield is on tomatoes, which increased by 232%, followed by beans (180%), maize (95%) and covo (*Brassica oleracea* var *acephala*) (59%). Montazar et al. (2020) and Postel et al. (2001) confirm the significance of drip irrigation in low-income communities and contend that it holds the key to alleviating a significant share of rural hunger and poverty. According to ZimVAC (2018), an average household size of six needs around 10 kg of cereal per month, translating into an annual cereal requirement of 120 kg per household. Prior to the drip intervention, the lowest household produced a minimum of 30 kg per annum compared to 100 kg per annum with drip intervention. The section ‘Impact of administering drip on crop yields’ presents the non-parametric tests contacted to determine the impact of administering the drip irrigation on crop yields.

#### Impact of administering drip on crop yields

This section measures the impact of administering drip irrigation on crop yields using the Mann–Whitney *U* test. The test compares the differences between two independent groups when the dependent variable is continuous or ordinal and non-normally distributed. The test is presented in Table 5 and hypothesises that

**TABLE 5:** Impact of administering a drip on crop yields.

Crop type	Dosage	*N*	Mean rank	Sum of ranks	*U*	*P*
Maize	Before drip	40	26	1026	205	0.000
	With drip	40	55	2215	-	-
	Total	80	-	-	-	-
Beans	Before drip	40	25	996	176	0.000
	With drip	40	56	2244	-	-
	Total	80	-	-	-	-
Covo	Before drip	40	25	1012	192	0.000
	With drip	40	56	2228	-	-
	Total	80	-	-	-	-
Tomatoes	Before drip	40	25	1007	186	0.000
	With drip	40	56	2234	-	-
	Total	80	-	-	-	-

Note: *P*-value significance level at 1%, 5% and 10%.

**H_0_**: The yield for maize, beans, covo and tomatoes is the same before the drip and after the drip intervention.

**H_1_**: The yield for maize, beans, covo and tomatoes is different before the drip and after the drip intervention.

The Mann–Whitney *U* test rejected the null hypothesis (H_0_) in favour of the alternative hypothesis (H_1_) across all the crop yields. There is thus enough evidence to suggest that administering the drip increases the yield across all crops. These findings are reinforced by the key informants from World Vision ENSURE, the custodians of the project who keep records and benchmark the output from the irrigation scheme. All the *P* values were highly statistically significant at a 1% level. These findings agree with those of Ararso et al. (2018) and Assefa et al. (2020); and for smallholder farmers, low-cost drip irrigation systems are a means through which they can maximise return on their crop yield by increasing the agricultural productivity per unit of land and through increased cropping intensity during the dry season. Further, Lozano et al. (2020), Sekondeko et al. (2017) and Dube et al. (2014) concur that if food stocks match or surpass the food requirements of a household, it is considered food secure. Additionally, Mhembwe et al. (2019) and Dube (2016) concluded that rural irrigation has a critical role in ensuring sustainable household and community income.

#### Impact of gender, age and education on crop production

This section measures the impact of gender, age and education on crop production using the Kruskal–Wallis *H* test of ranks. The test compares the differences between two independent samples of equal or different sizes. The test is presented in Table 6 and hypothesises that the mean ranks of the groups are the same.

**TABLE 6:** Impact of gender, age and education on crop production.

Gender	Maize	Beans	Covo	Tomatoes
Kruskal–Wallis *H*	3.423	0.082	2.040	0.090
df	1	1	1	1
Asymp. sig.	0.064	0.775	0.153	0.764
Age				
Kruskal–Wallis *H*	3.035	8.011	5.104	4.881
df	4	4	4	4
Asymp. sig.	0.552	0.091	0.277	0.300
Education				
Kruskal–Wallis *H*	2.666	0.000	1.164	0.674
df	1	1	1	1
Asymp. sig.	0.103	0.986	0.281	0.412

Note: *P*-value significance level at 1%, 5% and 10%.

df, degrees of freedom.

In all the analyses above, age, gender and education have no impact on crop production. Kruskal–Wallis is insignificant across all variables. This confirms that production across all these demographics is evenly distributed. Regarding education, all households are literate enough to apply the technical skills required in the administration of the irrigation scheme and crop production.

Consequently, regarding age, all household heads are within the economically active population, hence their contribution to the irrigation scheme is at most equal. Finally, regarding gender, all households had an equal chance of participation in the project.

### Qualitative analysis

This section provides a thematic analysis of open-ended questions from the semi-structured questionnaire administered to a census of 40 household beneficiaries and also the FGD outcomes of five key informants used in assessing the contribution of the drip irrigation scheme towards household food production. The thematic areas are drawn from both the 40 household respondents and the FGD and they include (1) the factors responsible for the success of the irrigation scheme, (2) the challenges of the irrigation scheme and (3) the potential strategies to improve household food production.

#### Factors responsible for the success of the irrigation scheme

The findings from the FGD corroborate the responses of the semi-structured interviews. Being the government agency, AREX is responsible for agricultural extension services in the country. In addition, AREX provides technical advice on water conservation and maintenance of the irrigation system as well as linking the irrigation users to the markets. All the key informants agreed on the important role of AREX services in educating farmers on the purchase of inputs (such as improved seed varieties, fertilisers and pesticides).

Apart from the institutional support, respondents affirmed to the following factors as key in the success of the irrigation scheme: adequate water supply (the availability of sufficient water from the catchment areas (such as dams), availability of inputs (such as short maturing and pest-resistant varieties), labour (such as cheap household labour and also hiring of local labour) and the availability of land (such as communal land, offered by the government through the district lands and agriculture). Other factors include distance to the scheme (on an average, it takes 45 walking minutes from the respondent’s residents to their plots). This implies an increase in crop yields emanating from time spent on the plot.

To cushion against management and maintenance problems, all beneficiaries make a monthly contribution of $30.00 towards the maintenance of the irrigation system. The maintenance, amongst other factors, includes repairing of damaged pipes. The beneficiaries explained that the money is used for the maintenance of the structure and repairing of damaged pipes as well as canal clearance to remove sediments and grass. In addition, a management committee was selected to decide on the schedule for water distribution, to plan and organise canal clearance and to resolve problems that might arise from the irrigation scheme.

The factors mentioned are congruent with Assefa et al. (2020) and Mozumdar’s (2012) findings. Mozumdar identified the availability of sufficient water resources, suitable land and labour, and institutional support as key factors to the success of irrigation systems.

#### Major challenges of the irrigation scheme

A number of challenges were identified as constraints to ensuring household food production through drip irrigation. The challenges include siltation of the dam and damage to water supply pipelines, as well as the drip irrigation pipe. For example, during the 2016/2017 rain season, some pipes were wiped away and some leaked. The informants revealed that, although drip irrigation is efficient and allows the maximum use of available water, it clogs holes in pipes, thus affecting the efficiency of the irrigation system. In addition, the beneficiaries face problems in accessing markets for selling surplus produce. The road is poorly serviced, with the nearest market 10 km away. More so, farmers grow the same types of crops at the same time, thereby flooding the market and, thus, are forced to dump their produce to traders at very low prices making little profits, especially on perishable goods such as tomatoes and vegetables which are difficult to stock.

The informants affirmed the beneficiaries’ claims that the lack of technical know-how threatens the functionality of the system. Narayanamoorthy and Devika (2018) and Mudimu (2003) reiterated that smallholder farmers lack knowledge, training and appropriate managerial skills to manage some of the technologies. This leads to inefficiencies, wastage of resources and technical failures of the provided equipment. Farmer education is necessary and the government should recruit more extension workers to educate farmers on modern technology so as to maximise their production activities.

These challenges identified by the informants have also been summed up in other areas that have been studied in Zimbabwe. For example, according to Mutiro and Lautze (2015), smallholder irrigation schemes in Southern Africa have consistently failed in their goal of improving rural livelihoods and sustainable agricultural production for food security and eradicating poverty. In Zimbabwe, the underachievement of smallholder irrigation schemes is largely because of diverse mutually reinforcing factors such as weak technical capacity, inadequate administrative arrangements and uncoordinated market mechanisms (Jacobs et al. 2013; Mhembwe et al. 2019; Moyo et al. 2017; Mujere et al. 2011).

In addition, some seasons experience very low rainfall. Reservoirs can also be too low towards the end of the dry season which results in water rationing. Water rationing, especially in the dry season, adversely affects crop production.

#### Potential strategies to improve household food production

With the aim of improving the benefits of the scheme, beneficiaries gave varied responses on strategies to improve food production using the drip irrigation scheme. The responses were based on the experiences and perceptions of both semi-structured questionnaires and the FGD. Most of the respondents in the semi-structured questionnaires (78%) expressed that promoting new technology acquisition in terms of methods and inputs is important in improving food production. This argument is similar to that made by Ye et al. (2019) and Goshu, Kassa and Ketema 2013) who argued that obtaining modified irrigation technologies into the hands of the beneficiaries is critical to intensifying crop production.

Moreover, both informants and beneficiaries indicated that increasing access to credit through micro-credit can help improve food production using improved seed varieties, fertilisers, pesticides and herbicides. This should be supported by regular ‘master farmer’ training programmes on the proper application, procedures and sustainable farming practices.

In another contention, beneficiaries have fewer choices when selling produce to the nearest market and there is a lack of reliable market information which reduces their profit margins. Hence, irrigation users felt the need for producer–retailer synergies as well as value-added support to increase the income proceeds from the scheme.

In addition, 89% of the respondents felt that the irrigated plot is too small to run as business beyond meeting food requirements; hence, they advocated for the expansion of the plot size per household. Respondents expressed that the community would be more food secure if the current irrigation scheme was extended to enable a household to irrigate more land. Respondents also emphasised increasing investment in local agriculture which shows the need to improve storage facilities to reduce post-harvest losses and improve the preservation of food stocks.

Most importantly, 92% of the respondents, in agreement with responses from the AREX officers indicated that improving water supply is crucial to food security, as it allows households to produce food all year round. The erratic rain supplies and depleted catchment sources during the dry season calls for an advanced method of water extraction, such as the solar-powered borehole irrigation systems which increase water availability. This allows continuous food production during peak and off-peak season(s).

## Conclusion

This study assessed the contribution of the small-scale drip irrigation scheme towards food production and nutritional outcomes in Burirano Ward 4, Chipinge, Zimbabwe. A mixed-method convergent parallel design was utilised, drawing from semi-structured questionnaires administered on a census of 40 household beneficiaries, and FGD of five key informants, directly linked to the irrigation scheme to explore three key questions. These questions included (1) To what extent has the drip irrigation scheme increased food production and nutritional outcomes? (2) What are the major factors affecting the effectiveness of the drip irrigation scheme? and (3) What are the possible strategies that can be adopted to increase food production, through the drip irrigation system? In response to Question 1, a quantitative analysis was conducted utilising non-parametric tests. Findings from the analysis showed that the drip irrigation scheme has positively contributed to household food production. In response to Questions 2 and 3, a qualitative thematic analysis was conducted on FGD and open-ended questions from the semi-structured questionnaires. Three key themes were discussed, namely, factors responsible for the success of the irrigation scheme, major challenges of the irrigation scheme and potential strategies to improve the household food production.

It was found that the factors responsible for the success of the Chidzadza World Vision ENSURE irrigation scheme are the availability of cheap labour, institutional support and the ease of access to irrigation plots. Through the drip irrigation scheme, the household food consumption/nutrition levels improved significantly. Apart from the occasional staple food production, households were able to produce adequate nutritious food such as covo, tomatoes and beans. In addition, households would practice animal husbandry using income from the horticultural proceeds and water from the irrigation catchment areas.

Major constraints to ensuring household food production and nutrition through the drip irrigation scheme include siltation of the dam, damage to water supply pipelines, frequent clogging of holes, difficulties in accessing markets as well as lack of technical know-how on the functionality of the system. Suggestions on improving the effectiveness of the scheme include continuous farmer training support, access to farming credit lines, the introduction of new water harvesting technologies as well as value addition and producer retailer marketing synergies. The study is, therefore, relevant as a policy strategy on encouraging the adoption of the drip irrigation scheme in dry rural areas, in addressing food insecurity. From the study findings, food availability is realised from the increase in yields or food production. Food accessibility is related to the availability of income from selling the farm produce. Consequently, food utilisation is realised from the nutritional component obtained from vegetables and legumes harvested from the plots. The food stability component is compromised because of a variety of challenges deterring the effectiveness of the irrigation system. Key in realising all the food security components is increased water supply from the catchment source. This can be enhanced through the construction of solar-powered borehole water systems.

## Footnotes

1 World Vision ENSURE Food Security Program is a World Vision led and USAID-funded 5-year intervention designed to sustainably impact 215,000 vulnerable and food-insecure Zimbabweans in Manicaland and Masvingo Provinces. The programme’s three strategic objectives include improving nutrition amongst women of reproductive age and children under the age of five; increasing household income via improved agriculture production and marketing; and increasing resilience to food insecurity of communities via improved disaster risk reduction and natural resource management.
